# The Effect of Topical Anesthesia on the Phonation Process During High-Speed Digital Imaging Laryngoscopy in Patients with Laryngopharyngeal Reflux: A Randomized, Double-Blind, Placebo-Controlled Crossover Trial

**DOI:** 10.3390/medicina62081505

**Published:** 2026-08-05

**Authors:** Mario Bilić, Juraj Slipac, Matko Prtorić, Maša Mitrović, Iva Botica, Lana Kovač Bilić

**Affiliations:** 1 Department of Otorhinolaryngology and Head and Neck Surgery, University Hospital Centre Zagreb, 10 000 Zagreb, Croatia; mbilic100@gmail.com (M.B.); jslipac@gmail.com (J.S.); iva.botica@yahoo.com (I.B.); 2 School of Medicine, University of Zagreb, 10 000 Zagreb, Croatia; matko.prtoric35@gmail.com (M.P.); masa.mitrovic296@gmail.com (M.M.)

**Keywords:** high-speed digital imaging, laryngoscopy, topical anesthesia, laryngopharyngeal reflux

## Abstract

*Background and Objectives*: High-speed digital imaging (HSDI) is the most advanced method of phonation process analysis. Hoarseness is the most common indication for HSDI, and laryngopharyngeal reflux (LPR) is an etiological cofactor of hoarseness in 50% of cases. Clinical practice in HSDI includes the elective use of topical anesthesia (TA), but without defined recommendations and indications for use, and a potential altering effect of TA on the phonation process has not been unequivocally proven. The aim of this prospective, randomized, double-blind, placebo-controlled study was to examine the effect of TA application on the phonation process by analyzing Voice-Vibratory Assessment with Laryngeal Imaging (VALI) form parameters in subjects with LPR. *Materials and Methods*: A total of 70 volunteer subjects were included in the study; 50 subjects who completed both recording sessions were included in the final analysis. All subjects were recorded in two modalities, with TA application (lidocaine spray 100 mg/mL) and without it, on two consecutive days. *Results*: By evaluating phonation process parameters according to the VALI form, we determined that TA application during HSDI in subjects with LPR was not associated with statistically significant changes in the evaluated phonation process parameters. *Conclusions*: TA application during HSDI in subjects with LPR was not associated with significant changes in the analyzed phonation process parameters. Further research is needed to determine the benefit of TA in reducing pain and discomfort during examination, weighed against the risk of potentially life-threatening side effects, in order to individualize the approach to each patient.

## 1. Introduction

Phonation is a complex process of voice and speech production that enables expression, acoustic transmission of messages, information, and emotions and represents the foundation of verbal communication. The vocal folds are unique endolaryngeal structures that extend horizontally through the larynx and are the most important anatomical structures for phonation due to their specific architecture, which allows the conversion of aerodynamic energy into acoustic waves. The phonation process involves the interaction of expiratory airflow pressure that, as it passes between the vocal folds, causes their displacement and vibration, forming a mucosal wave and generating acoustic waves that are subsequently modified by the articulator and resonator system, producing the phenomenon of voice [1].

Methods of phonation analysis are numerous and are divided into visualization and non-visualization methods, depending on whether the vocal folds are visualized during analysis [1]. Non-visualization methods for evaluating the phonation process include the following techniques: auditory or perceptual evaluation, acoustic analysis, aerodynamic function measurement, and patient self-assessment of voice characteristics. Aerodynamic phonation tests determine the expiratory volume during phonation, analogous to respiratory volume measurement. The simplest clinical test for measuring vocal fold function during phonation is the determination of maximum phonation time of a vowel. Maximum phonation time (MPT) is assessed based on prolonged phonation of the vowel /a/ (measured in seconds using a stopwatch), following a maximum inhalation, at an optimal, spontaneous pitch and voice intensity. Acoustic voice analysis includes objective measurement of acoustic parameter values in voice recordings using acoustic voice analysis programmes (PRAAT, Multi-Dimensional Voice Programme MDVP). The most common acoustic parameter measures are fundamental frequency (F0), jitter, shimmer, and intensity. F0 is defined as the number of vibration cycles per second and is perceptually experienced as pitch. Intensity is determined by the rate of the glottal volume velocity and the resonant structures of the vocal tract. It is perceived as the loudness of the voice. It is measured on a logarithmic decibel scale (dB) [2].

Visualization of the larynx is essential in the diagnosis of phonation disorders. The clinical purpose of laryngeal visualization is to determine the etiology of the disorder, define the behavioural pattern and extent of impairment of vocal fold phonatory function, assess the patient’s prognosis, and establish a treatment plan [3]. Although clinicians have access to numerous diagnostic techniques for directly or indirectly assessing vibratory function, only visualization of the vocal folds provides a detailed insight into tissue condition, flexibility, and vibratory capacity. Outpatient laryngoscopy methods include indirect laryngoscopy, flexible transnasal laryngoscopy, and rigid transoral laryngoscopy. The frequency of vocal fold vibration during speech phonation exceeds the perceptual capacity of the human eye (males: 100–180 Hz; females: 180–220 Hz), and for adequate assessment of phonation—particularly in patients with discrete local findings or pathological changes not detectable by other visualization methods—vocal fold movements must either be slowed using stroboscopic technique or equipment capable of high-frequency sampling must be used. In clinical practice, detailed vibratory pattern examination of the vocal folds is performed using stroboscopy and a high-speed camera [4,5].

High-speed digital imaging (HSDI) laryngoscopy is the most advanced method of phonation process analysis, having revolutionized laryngeal visualization and enhanced understanding of vocal fold movement dynamics during phonation [6]. By capturing sequential photographs of the vocal folds at a recording frequency of 4000–20,000 Hz during phonation, it allows detailed and precise insight into the biomechanics of vocal fold movement and enables more accurate functional assessment of the pathophysiology of voice disorders, resulting in improved diagnosis and treatment of laryngeal pathological changes. In clinical practice, 4000 Hz is the standard frequency applied, while 2000 Hz is considered the minimum for quality analysis [7]. These characteristics ensure the capture and sampling of multiple photographs within a single glottal cycle (10–20 depending on the phonation frequency; a minimum of 16 for quality analysis), as well as continuous recording of multiple successive glottal cycles [8]. For habitual phonation, quality analysis requires a minimum of 1000 clear images of the full length of the vocal folds during the phonation phase [9]. This enables true analysis of vocal fold movement characteristics within a single cycle and across multiple glottal cycles (inter-cycle and intra-cycle variations). Currently, no clearly defined standards, practical guidelines for interpretation of findings, physiological parameter values, consensus for identifying all analyzed parameters and defining their values exist in the literature [8,10,11]. Recording evaluation can be performed through clinical subjective assessment of parameter characteristics or through software-generated secondary data allowing objective measurements (digital kymography) [12].

Laryngopharyngeal reflux (LPR) is a disease caused by retrograde return of gastric contents (gastric acid and pepsin) into the larynx and pharynx. The laryngeal mucosa is, due to a lack of protective mechanisms, sensitive to contact with acid and gastric enzymes, which causes mucosal damage and development of local inflammation [13,14]. It is estimated that 8–20% of the population suffers from LPR, and that up to half of all cases of hoarseness are associated with LPR [15]. The most common complaints reported by patients are globus pharyngeus, chronic dry irritative cough, frequent throat clearing, difficulty swallowing, and hoarseness. There are several complementary diagnostic approaches for LPR. For diagnosis based on history, the Reflux Symptom Index (RSI) questionnaire has been developed, which is completed by the patient who grades their symptoms, with a result > 13 considered positive. Diagnosis based on laryngoscopy relies on the Reflux Finding Score (RFS) form, completed by the clinician who grades local examination parameters, with a sum > 7 considered a pathological finding [16,17]. Diagnosis is established retrospectively, based on symptom reduction following regular use of proton pump inhibitors (PPI) for 2 to 3 months [18]. A 24 h dual intraluminal pH-metry diagnoses LPR by quantifying the number and duration of pH changes in the larynx and represents the most advanced method of LPR diagnosis [19].

Topical anesthesia (TA) is defined as a superficial loss of sensation in the conjunctiva, mucosa, or skin, produced by direct application of local anesthetic solutions, ointments, gels, or sprays. Various active substances are available for use, the most commonly lidocaine [20]. In otorhinolaryngology, it is applied during tympanocentesis, myringotomy, transtympanic administration of gentamicin or steroids, transnasal or transoral endoscopy of the nasal cavity, pharynx, and larynx [21,22]. Standard clinical practice for rigid transoral laryngoscopy, as well as for HSDI, includes the elective use of topical anesthesia of the oropharynx and oral cavity, but without clearly defined recommendations and indications for use [23]. By temporarily blocking the conduction of nerve impulses from sensory and motor neurons at the site of application, the aim is to reduce the level of discomfort during the examination, suppress the gag reflex, and increase the possibility of adequate laryngeal visualization [24]. Results of conducted studies are not in agreement regarding the effect of TA application during flexible and rigid laryngoscopy on reducing discomfort and pain levels. Upon application of anesthetic to the oral cavity and oropharynx, there is a possibility of anesthetic draining into the larynx, which may consequently cause desensitization of the mucosa and affect the sensorimotor aspects of laryngeal function (phonation, swallowing, aspiration protection). There are disagreements about the potential altering effect of TA application, both on laryngeal function itself and on the endolaryngeal findings, which may have negative implications for interpretation of findings, resulting in misdiagnosis and consequently altering the treatment plan. Studies on the effect of applied TA during examination on the phonation process are scarce, and results are contradictory.

We hypothesized that topical anesthesia would produce no clinically or statistically significant change in phonatory parameters, as assessed by the VALI form, in patients with LPR.

## 2. Materials and Methods

This was a prospective, randomized, double-blind, placebo-controlled, two-period crossover trial with a 1:1 allocation ratio between the two sequence groups. No important changes were made to the study methods after trial commencement. Trial Registration: This trial was registered at ClinicalTrials.gov (Identifier: NCT07696429; University Hospital Centre Zagreb Protocol Record Class No. 8.1-19/31-2; registered 9 July 2026).

The aim of the study was to determine the effect of TA application on the results of phonation process analysis during HSDI laryngoscopy in subjects with LPR, by comparing phonation process parameters with and without TA application. A total of 70 male and female subjects aged 18–45 years with newly diagnosed LPR were enrolled in this prospective, randomized, double-blind, placebo-controlled study.

The study included subjects who had not previously undergone HSDI recording, who tolerated HSDI recording without TA, and who had newly diagnosed LPR.

Subjects with known allergy to lidocaine and/or other allergens, subjects with a resolved acute respiratory tract infection within a period of less than one month, smokers, regular consumers of alcoholic beverages, subjects with chronic rhinosinusitis, subjects with a history of surgery and/or radiation therapy of the larynx, neck, or thyroid gland, and subjects with newly diagnosed LPR in whom symptom reduction and improvement of local findings had not occurred after three months of regular proton pump inhibitor use were excluded from the study. A detailed medical history was taken from all subjects, including past diseases and habits (data on alcohol consumption, smoking, medication use, allergies, surgeries and/or radiation therapy of the larynx, neck, and thyroid). Reflux Finding Score was determined by fiberendoscopic examination according to the applicable classification. Subjects completed the RSI questionnaire. Only subjects with RSI > 13 and RFS > 7 were included in the study.

A computer-generated random number was assigned to each subject, which determined the order of recording modalities (subjects with even numbers were first recorded with TA, and subjects with odd numbers were first recorded without TA). Simple randomization was used; no blocking or stratification was applied. Allocation was concealed using sequentially numbered, sealed instruction sheets prepared in advance and opened only by the administering physician immediately before each session. Once the study started, the order of recording modalities could not be changed, and in further data processing, only parameters from subjects recorded in both modalities were used. Randomization and the order of testing were unknown to the examiner. Application of anesthetic or physiological saline to the oral cavity and oropharynx, including the base of the tongue, posterior pharyngeal wall, and soft palate, was performed by a junior physician behind closed doors, without knowledge of the substance used. Lidocaine in spray form was used in the study (concentration 100 mg/mL; 0.1 mL of lidocaine per actuation; BELUPO lijekovi i kozmetika d.d., Koprivnica, Croatia). TA or physiological saline (B. Braun Melsungen AG, Melsungen, Germany) was applied with one actuation to each of the stated anatomical regions. Topical lidocaine and placebo were administered using identical spray devices and identical application procedures to maintain blinding. HSDI recording was performed 5 min after TA application.

Each subject underwent HSDI recording on two consecutive days (a sufficiently short period in which phonotrauma, hormonal and health changes in subjects are not expected) in two different, aforementioned recording modalities. The two examination sessions were separated by approximately 24 h; since the pharmacological effect of topical lidocaine lasts less than one hour, this interval was considered sufficient to eliminate residual pharmacological effects, and a clinically relevant carry-over effect between periods was therefore considered unlikely.

Examinations and HSDI recordings were performed by an experienced otorhinolaryngologist, subspecialist phoniatrician with more than five years of work experience. All examinations were performed in the outpatient phoniatrics clinic of the Department of Otorhinolaryngology and Head and Neck Surgery, University Hospital Centre Zagreb, Croatia. The Wolf 5562 HRES ENDOCAM rigid 90° endoscope with the Wolf Auto LP 5132 Hlight light source was used (RICHARD WOLF GmbH, Knittlingen, Germany). HSDI was performed under outpatient conditions in an otorhinolaryngological examination chair in which the subject sat upright, with mouth open and tongue protruded, held by the examiner with gauze. This was followed by gentle insertion of the endoscope into the oral cavity and pharynx, preferably without contact with the mucosa, passage along the midline of the upper tongue surface along the glossoepiglottic fold over the upper surface of the epiglottis with tilting of the endoscope to achieve visualization of the full length of the vocal folds. At the moment of vocal fold visualization, centering and focusing of the image was performed, and the subject was asked to phonate the vowel /a/ dynamically and frequently in a relaxed manner, whereupon the examiner activated the HSDI recording for a duration of two seconds. Following the recording, the recording was reviewed, and its quality adequacy was assessed (minimum 1000 clear, focused consecutive images showing the full length of the vocal folds and other laryngeal structures during the steady phonation phase). In cases where recording quality was found to be inadequate, the recording was repeated. The frequency (Hz) and phonation intensity (dB) were recorded using the integrated microphone of the HSDI system and processed with the accompanying software (RICHARD WOLF GmbH, Knittlingen, Germany). MPT was determined by selecting the longest duration of dynamically and frequency-stationally relaxed phonation of the vowel /a/ in a single breath, measured in seconds from a total of 3 measurements with a stopwatch immediately before and after HSDI recording.

Recording evaluation was performed through subjective clinical assessment of parameter characteristics [12]. For the purpose of standardization, objectification, and unification of analysis, the Voice-Vibratory Assessment with Laryngeal Imaging (VALI) form was used, along with the European Laryngological Society (ELS) form for analyzing the glottal-closure pattern during the initial phases of the glottal cycle, as well as a modified classification of glottal insufficiency [25,26]. Analysis of each HSDI recording was performed by two experienced otorhinolaryngologists, subspecialists in phoniatrics with more than five years of work experience, using visual–perceptual assessment. After completing the assessment of the HSDI recording, they presented their assessment findings. In case of discrepancies in the assessed parameter values, a joint re-analysis and verbal discussion followed until consensus on the parameter value was reached, after which the next recording was analyzed. The RFS was re-determined from the recording according to Belafsky et al. [16]. Following the ELS recommendation, we also analyzed the glottal-closure pattern during the phonation initiation process and determined the type of glottal insufficiency according to the ELS classification.

A follow-up examination of subjects from the LPR group and re-determination of RFS by fiberscopic examination and RSI by questionnaire completion was performed within three months of study participation, during which subjects regularly took PPI therapy and adhered to hygienic-dietary measures. In cases of persistent pathological RFS and RSI findings, subjects were excluded from the study.

The pre-specified outcome measures, all considered of equal (primary) importance, were the VALI-form phonatory parameters (glottal sufficiency pattern, mucosal wave amplitude, free-edge amplitude, vertical level, non-vibrating segment, supraglottic activation, free-edge contour, open/closed phase ratio, phase symmetry, periodicity, and axis shift), ELS glottal-closure classification, RFS, maximum phonation time, fundamental frequency, and intensity, each compared with versus without topical anesthesia. No hierarchy of primary and secondary outcomes was pre-specified. These outcome measures remained unchanged throughout the study.

### Statistical Analysis

Because of the crossover design, each participant served as his or her own control; comparisons between treatment conditions were therefore performed using paired statistical tests. No formal analysis of period effects was performed because no learning effect or residual treatment effect was expected between the two examination sessions. No subgroup or covariate-adjusted analyses were pre-specified or performed.

The collected data were analyzed and graphically displayed. The Kolmogorov–Smirnov test was applied to test the normality of distributions of quantitative characteristics. Descriptive statistics were used to describe the distributions of these variables. Normally distributed variables were described using means and standard deviations, while variables that do not follow a normal distribution were described using medians and ranges. Testing of differences in measured characteristics with and without TA application was performed using the parametric paired *t*-test or the nonparametric Wilcoxon test, depending on the normality of their distribution. Spearman correlation and regression analysis were applied in the analysis of associations and prediction of anatomical structures and measured characteristics. Values of *p* < 0.05 were considered statistically significant. Statistical analyses were performed using IBM SPSS Statistics for Windows (IBM Corp., Armonk, NY, USA). Multiple phonatory parameters were analyzed, including VALI parameters, MPT, F0, intensity, and RFS; therefore, the findings should be interpreted with consideration of the number of statistical comparisons performed.

## 3. Results

**Figure 1:**
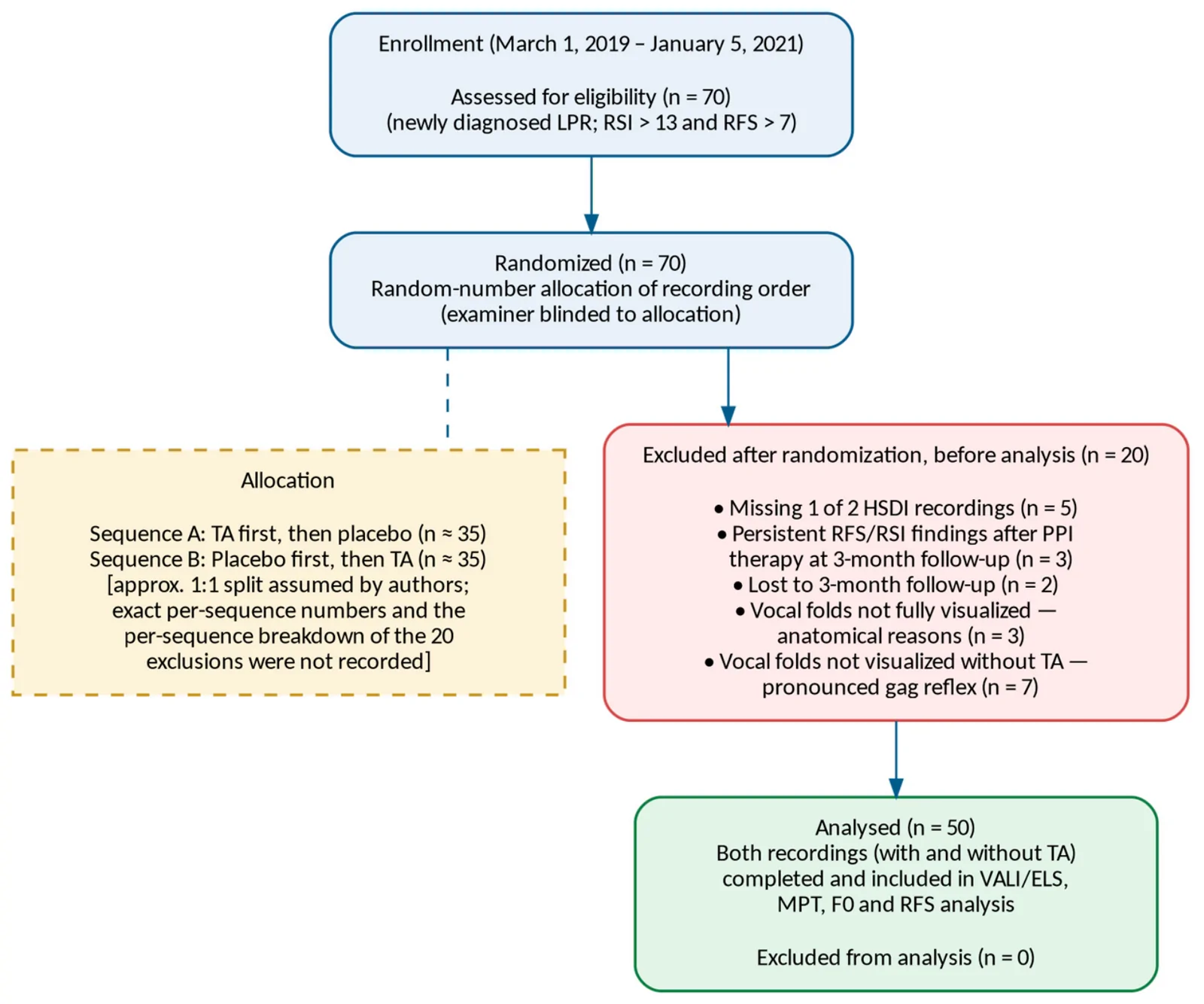
Flow diagram.

A total of 70 subjects were enrolled in the study, of whom only 50 met the inclusion and exclusion criteria (Figure 1). The mean age was 23.6 ± 7.02 years. There were 17 (34%) male and 33 (66%) female subjects. A total of 20 subjects were excluded from the study. Five subjects were excluded due to inadequate processing (absence of two HSDI recordings on two consecutive days), three subjects were excluded due to stationary local findings and/or persistence of LPR symptoms following PPI therapy, and two subjects were excluded due to inadequate follow-up (absence of a follow-up examination three months after recording for evaluation and confirmation of LPR diagnosis). Inability to visualize the full length of the vocal fold during two seconds of recording due to anatomical reasons was observed in three subjects. In seven subjects, due to a pronounced gag reflex, it was not possible to visualize the vocal folds without applied TA. Inability to adequately perform the examination, to visualize the full length of the vocal fold during two seconds of the examination, was noted in the aforementioned 10 subjects, who were excluded from the study. Data from these 20 subjects were not statistically analyzed. 

Recruitment ran from 1 March 2019 to 5 January 2021 (primary completion), with study completion (final data processing) on December 5, 2021. The trial was completed according to the study protocol without early termination. Of the 70 randomized subjects, an approximately equal number were allocated to each recording sequence (topical anesthesia first vs. placebo first); exact per-sequence numbers, and the per-sequence breakdown of the 20 post-randomization exclusions listed above, were not recorded separately. Analysis was restricted to the 50 subjects who completed both recording sessions and the 3-month follow-up (per-protocol analysis); an intention-to-treat analysis was not performed because phonatory parameters cannot be measured for incomplete sessions.

HSDI recording analysis of the phonation process was performed according to the VALI form. The following parameters were analyzed: glottal sufficiency pattern, mucosal wave amplitude, free edge amplitude, vertical level of the vocal folds, non-vibrating segment of the vocal fold, supraglottic structure activation, free edge contour, open-to-closed phase ratio of the glottal cycle, phase symmetry of the vocal folds, periodicity, and axis shift in the vocal folds during the phonation process. The ELS form was used to assess the glottal-closure pattern and type of glottal insufficiency.

**Figure 2:**
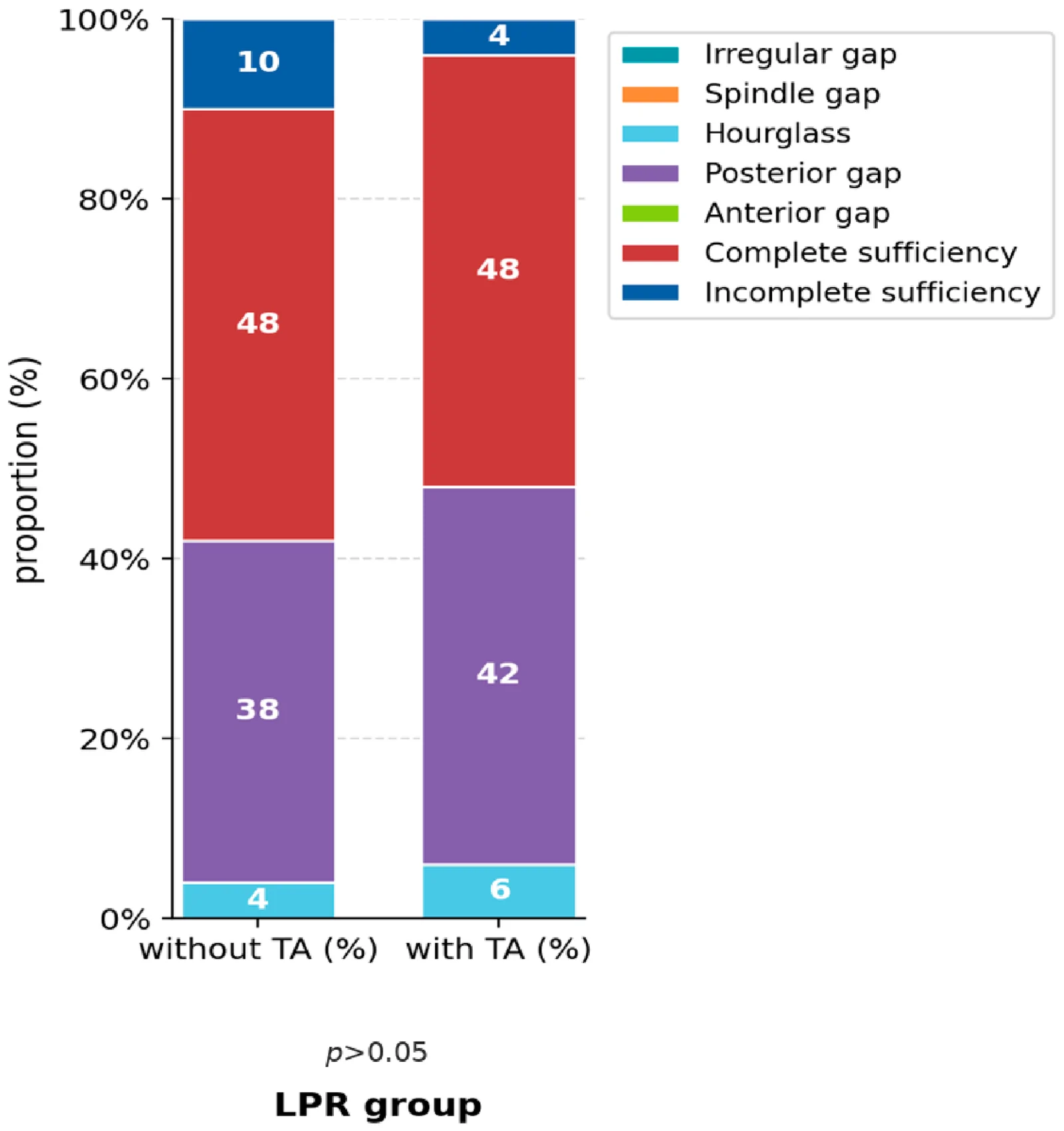
Distribution of glottal sufficiency patterns in the LPR group of subjects with and without applied TA; *Wilcoxon test, *p* < 0.05.

The most common glottal sufficiency pattern in the LPR group without and with applied TA was complete glottal sufficiency (48% of subjects with TA and 48% without TA). No statistically significant differences in glottal sufficiency patterns were detected between examinations with and without TA application in LPR subjects (*p* > 0.05) (Figure 2).

The median mucosal wave amplitude of both vocal folds was 60% (range 0–100%); after TA application, it was 60% (range 0–100%). The change was also not statistically significant (*p* > 0.05).

Analysis revealed no statistically significant change in mucosal wave amplitude following TA application in either subject group. Values of mucosal wave amplitudes of the LPR subject group are presented in Table 1.

TA application had no effect on the change in vocal fold vertical level. All subjects with and without TA had vocal folds at the same vertical level (LPR subject group: *p* > 0.05).

No effect of TA application on the change in the proportion of the non-vibrating vocal fold segment was observed. The median non-vibrating segment without application was 20% (range 0–60%) (*p* > 0.05). After TA application, the median non-vibrating segment was 20% (range 0–60%), (*p* > 0.05).

Data analysis revealed no statistically significant change in anteroposterior and laterolateral activation of supraglottic laryngeal structures. With and without TA application, the median activation of laryngeal suprastructures was zero (range 0–2), with no change after TA application (LPR subject group: *p* > 0.05).

A concave free edge contour was observed in 4% of subjects, while the remaining 96% had a straight free edge contour of both vocal folds. TA application did not statistically significantly change the free edge contour (*p* > 0.05).

**Figure 3:**
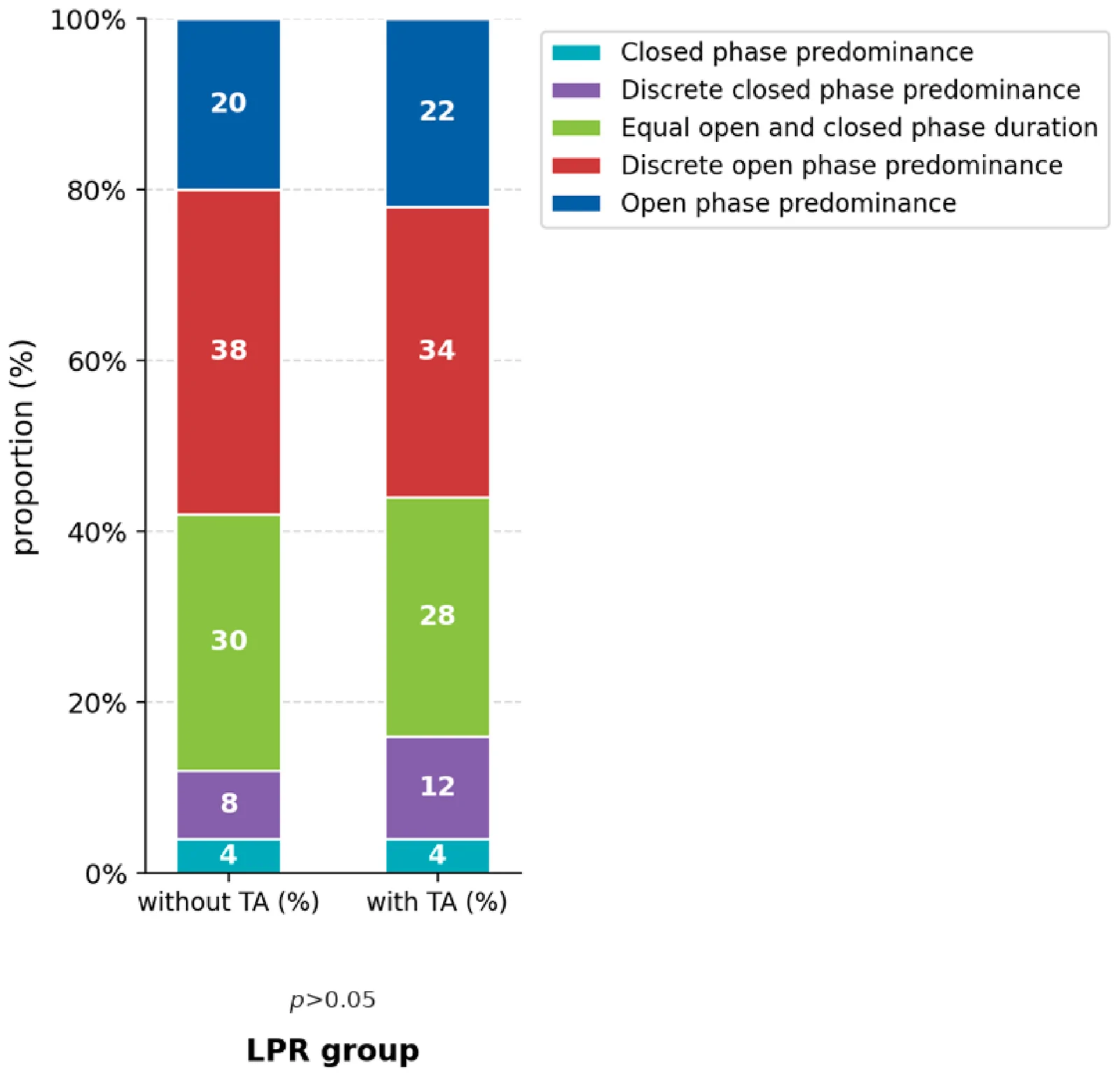
Distribution of open-to-closed phase ratio categories of the glottal cycle in the LPR subject groups with and without applied TA; *Wilcoxon test, *p* < 0.05.

The majority of subjects (38%) had a discrete predominance of the open phase of the glottal cycle. TA application, without statistical significance, reduces the proportion of subjects with discrete open phase predominance and increases the proportion of subjects with discrete closed phase predominance (*p* > 0.05) (Figure 3).

The median phase symmetry was 100% (range 20–100%). TA application did not statistically significantly change phase symmetry; the median was 90% (range 20–100%) (*p* > 0.05).

TA application did not statistically significantly change periodicity (*p* > 0.05). Without TA application, the median periodicity was 100% (range 80–100%). After TA application, the median was 100% (range 90–100%) (*p* > 0.05).

**Figure 4:**
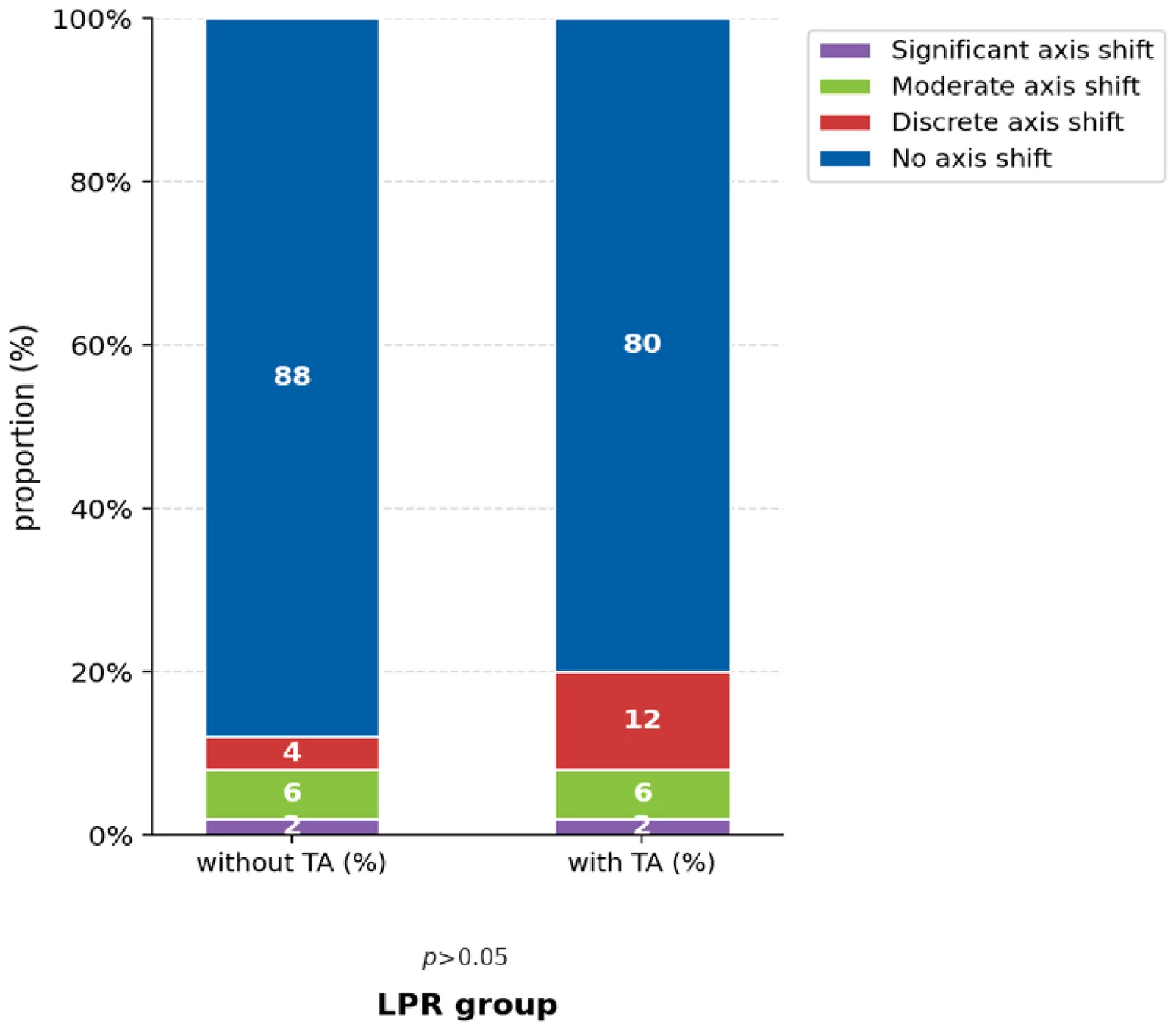
Distribution of vocal fold axis shift categories in the LPR subject groups with and without applied TA; *Wilcoxon test, *p* < 0.05.

TA application in the LPR subject group reduces the proportion of subjects without axis shift in the vocal folds during phonation by 8% and increases the proportion of subjects with discrete shift by 8%. The changes are not statistically significant (*p* > 0.05) (Figure 4).

**Figure 5:**
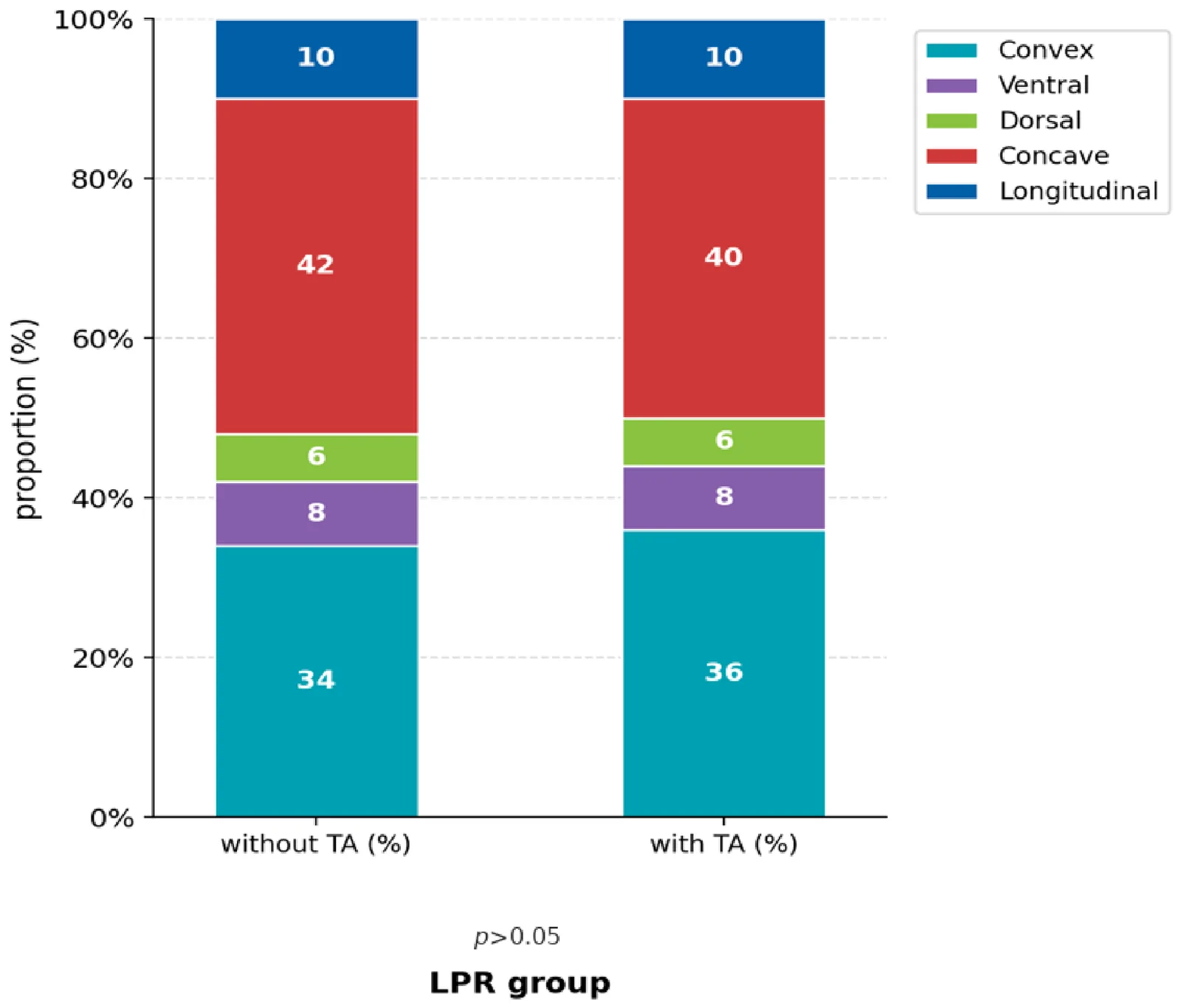
Distribution of glottal-closure patterns in the LPR subject groups with and without applied TA; *Wilcoxon test, *p* < 0.05.

The most common glottal-closure patterns were concave (40% and 42% of subjects) and convex (36% and 34% of subjects). TA application reduces the proportion of concave glottal-closure patterns and increases the proportion of convex patterns, while changes in the proportions of other closure patterns are not observed. The changes are not statistically significant (*p* > 0.05) (Figure 5).

**Figure 6:**
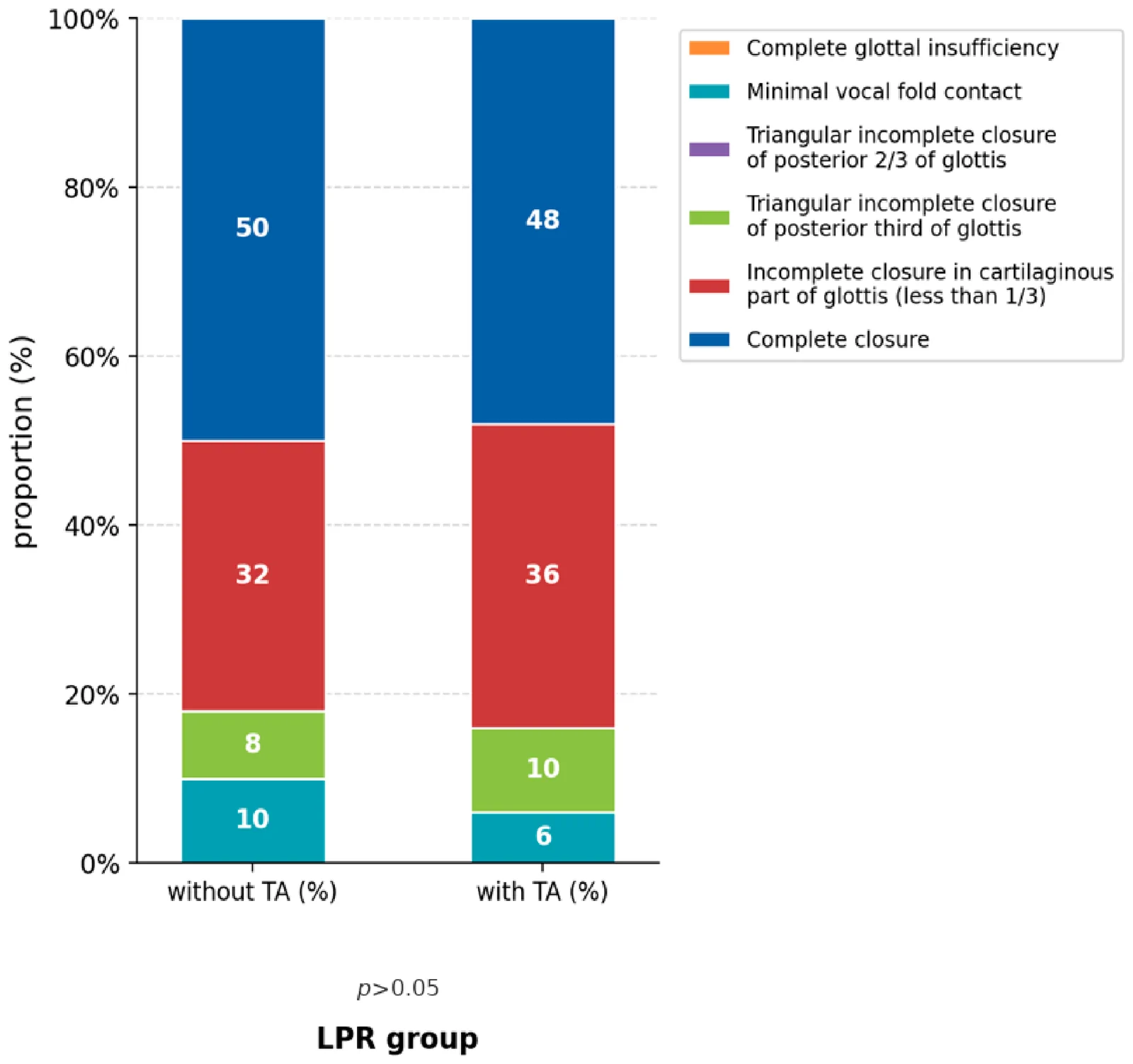
Distribution of glottal insufficiency types in the LPR subject groups with and without applied TA; *Wilcoxon test, *p* < 0.05.

Complete glottal closure and incomplete closure in the cartilaginous part of the glottis (less than 1/3) are the most common types of glottal insufficiency during phonation according to the ELS classification in the LPR subject group (50% and 32% of subjects). TA application produced no statistically significant change in the type of glottal insufficiency in subjects with LPR (*p* > 0.05) (Figure 6).

The median MPT before the examination was 20 s (range 16–51 s), after HSDI without TA it was 20 s (range 15–50 s), while after TA application it was 19 s (range 15–45 s). The changes were not statistically significant (*p* > 0.05; *p* > 0.05). The median phonation frequency during HSDI without TA was 184 Hz (range 80–302 Hz); with TA it was 187 Hz (range 88–302 Hz). The change was not statistically significant (*p* > 0.05).

This was a pre-specified additional analysis. Comparison of RFS results from fiberoptic flexible transnasal laryngoscopy with rigid HSDI laryngoscopy without TA application showed no statistically significant change in RFS (*p* > 0.05). The mean RFS value by fiberoptic laryngoscopy was 8.76 ± 1.36. By HSDI laryngoscopy without TA, it was 8.98 ± 1.34. RFS with TA application was 9.04 ± 1.59. The difference was not statistically significant (*p* > 0.05).

No adverse events or complications related to topical lidocaine or placebo administration were observed during the study.

## 4. Discussion

A review of the available literature established that, to date, no study has been conducted on the effect of TA application on the results of phonation process analysis during HSDI laryngoscopy. Also, unlike other visualization methods (rigid transoral laryngoscopy/flexible transnasal laryngoscopy with or without stroboscopy), the light source for HSDI is warm (xenon lamp) and uses an open air-cooling system at the tip of the endoscope during use, so results of studies conducted for other phonation analysis methods can only be applied to HSDI to a limited extent [2].

The aim of this study was to determine the effect of TA application on numerous phonation process analysis parameters during HSDI in subjects with LPR, with the goal of optimizing HSDI performance and standardizing scientific research using HSDI.

Results of previously conducted studies do not agree on the possible altering effect of applied TA on laryngeal function and endolaryngeal findings, which may have negative implications for the interpretation of findings and result in misdiagnosis. Studies on the effect of TA application on the phonation process are scarce, and results are contradictory. Peppard et al., comparing videostroboscopic phonation parameters during flexible laryngoscopy in healthy subjects with and without TA application, demonstrated no statistically significant changes in mucosal wave amplitude, amount of laryngeal secretions, supraglottic structure activation during phonation, or significant changes in subjects’ vocal range [27]. Jacobs et al., using acoustic voice analysis in healthy professional sopranos during flexible laryngoscope and TA use, did not demonstrate statistically significant changes in voice quality and range, explaining this by the subjects’ excellent singing technique compensating for TA effects [28]. Maxwell et al., using electrolaryngography and flexible laryngoscopy in healthy subjects, did not demonstrate statistically significant changes in electrolaryngogram findings after TA application and endolaryngeal presence of the endoscope [29]. Yang et al. did not demonstrate statistically significant changes in acoustic voice analysis parameters in healthy subjects, while with rigid laryngoscope use they obtained statistically significant changes in jitter, shimmer, and F0 [30]. Hu et al., using acoustic voice analysis in healthy subjects, demonstrated a statistically significant increase in jitter following TA application [31]. The difference between these findings and our results may be related to methodological differences, as jitter in the study by Hu et al. was assessed using a non-invasive acoustic voice analysis technique based on microphone recordings. Additionally, the presence of LPR-related mucosal changes in our study population may have influenced laryngeal sensitivity and potentially affected the response to topical anesthesia. Lim et al. statistically significantly demonstrated a reduction in minimum phonation frequency, a reduction in MPT, and an increase in phonation F0 following TA application during flexible laryngoscopy in healthy subjects [32]. These differences may be partially explained by variations in the examination technique, as flexible laryngoscopy is performed transnasally, whereas HSDI in our study was performed using an oral approach. Furthermore, our study included patients with LPR, and chronic laryngeal inflammation associated with this condition may have influenced mucosal sensitivity and the effects of topical anesthesia. Furthermore, Lester et al. demonstrated a reduction in nasal cavity discomfort following TA application with changes in swallowing act characteristics and increased aspiration potential, evaluated by flexible endoscopy, explaining the results by the passage of anesthetic into the oropharynx and laryngopharynx and consequent change in laryngopharyngeal function [21].

In most of the cited studies, physiological saline was used as placebo in the control group, while the control groups of other studies received no substances. The results of this study show that TA application did not statistically significantly change F0 and phonation intensity during the examination. TA application, as well as the HSDI examination itself, did not statistically significantly change subjects’ MPT. Analysis of HSDI recordings according to the VALI form revealed no statistically significant change in analyzed phonation process parameters (glottal sufficiency pattern, mucosal wave amplitude, free edge amplitude, vocal fold vertical level, non-vibrating vocal fold segment, supraglottic structure activation, free edge contour, open-to-closed phase ratio of the glottal cycle, vocal fold phase symmetry, periodicity, and vocal fold axis shift) following TA application.

This is the first study in which laryngeal secretion characteristics were analyzed by HSDI with phonation recording at a frequency of 4000 Hz. The study included, to date, the largest number of subjects and was conducted in a group with unified pathology with placebo application. Potential hormonal changes and changes in subjects’ health status were eliminated by the short period between two recordings and by the selection of subject age (<45 years).

Vocal fold visualization and RFS determination, along with RSI determination, are the basis of LPR diagnosis. According to Belafsky et al., LPR can be diagnosed with 95% certainty in subjects with an RFS result > 7 [16]. Results of the Milstein et al. study, conducted in a healthy subject population, show differences in detecting LPR signs depending on the laryngeal visualization technique, using ENT/GER normal signs. They concluded that fiberoptic transnasal laryngoscopy, compared to rigid transoral laryngoscopy, has greater sensitivity and lower specificity in identifying signs of laryngeal irritation and LPR signs in healthy subjects [33]. Eller et al., in their study conducted in subjects with LPR, concluded that although there are differences in the values of individual RFS parameters determined by rigid, flexible, or distal-chip laryngoscopes, the total RFS does not change significantly depending on the laryngeal visualization technique [34]. Results of this study show that RFS did not differ significantly depending on the method of determination (flexible laryngoscopy/HSDI). It is important to note that in this study we did not analyze potential changes in individual RFS parameter values, but only the total RFS. HSDI-determined RFS with and without applied TA was not associated with statistically significant differences in the LPR subject group.

A limitation of this study is that physiological saline was used as a placebo rather than a true placebo with organoleptic properties matching those of the lidocaine solution. Lidocaine may produce recognizable sensory effects, such as a bitter taste and mucosal numbness, which could potentially compromise participant blinding. The adequacy of blinding was not formally assessed by asking participants to identify the administered solution (lidocaine versus saline); therefore, the success of blinding cannot be confirmed. However, in the vast majority of previous studies, physiological saline was used as a placebo, while in the remaining studies no substance other than topical anesthesia was applied. Only one study described the use of an amaranth solution (*Amaranthus caudatus* L.), although the preparation methodology and solution concentration were not clearly reported.

This study has several additional limitations that should be acknowledged. Although the study included 50 participants and employed a randomized, double-blind, placebo-controlled crossover design, the relatively small sample size and the absence of an a priori sample size calculation may have limited the statistical power to detect subtle differences in individual phonatory parameters. In addition, interrater reliability between the two phoniatrists who performed the VALI assessments was not formally evaluated. Although all disagreements were resolved by consensus, the lack of statistical assessment of interrater reliability should be considered a limitation of this study.

Furthermore, patient-reported outcomes regarding discomfort during the examination and gag reflex intensity were not assessed, as they were not included among the predefined VALI-based outcomes. The absence of these subjective measures represents a limitation, as they could have provided additional information regarding the tolerability of HSDI examination and the potential clinical benefit of topical anesthesia.

Participants who were unable to tolerate HSDI examination without topical anesthesia because of a pronounced gag reflex were excluded from the final analysis. Consequently, this may have introduced selection bias, and the findings may not be fully generalizable to patients who require topical anesthesia to successfully undergo HSDI examination, as this subgroup may derive the greatest clinical benefit from its use.

Further research is needed to determine the benefits of TA application for reducing pain and discomfort levels during examination, as well as the potential effect on the difficulty level of examination performance, bearing in mind the risk of developing potentially life-threatening side effects of applied TA. This would individualize the approach to each patient and achieve better and safer healthcare.

## 5. Conclusions

Based on the results obtained in this study, we determined that TA application during laryngoscopy and HSDI phonation recording was not associated with statistically significant differences in the analyzed HSDI phonation recording parameters, as evaluated using the VALI form, in subjects with LPR.

## Figures and Tables

**Table 1:** Mucosal wave amplitude values of both vocal folds in the LPR subject groups with and without TA application, *Wilcoxon test, *p* < 0.05.

	Mucosal Wave Amplitude	Median (%)	Minimum (%)	Maximum (%)
LPR subjects	Right vocal fold without TA	60	0	100
	Right vocal fold with TA	60	0	100
	Left vocal fold without TA	60	0	100
	Left vocal fold with TA	60	0	100

## Data Availability

The data presented in this study are available on request from the corresponding author. The data are not publicly available due to privacy restrictions.

## Abbreviations

The following abbreviations are used in this manuscript:

HSDI	High-Speed Digital Imaging
LPR	Laryngopharyngeal Reflux
TA	Topical Anesthesia
VALI	Voice-Vibratory Assessment with Laryngeal Imaging
ELS	European Laryngological Society
RFS	Reflux Finding Score
RSI	Reflux Symptom Index
MPT	Maximum Phonation Time
PPI	Proton Pump Inhibitor
F0	Fundamental Frequency
